# Endometriosis in adolescence: A long-term follow-up fecundability assessment

**DOI:** 10.1186/1477-7827-3-14

**Published:** 2005-04-21

**Authors:** Gary Ventolini, Gary M Horowitz, Ronald Long

**Affiliations:** 1 Department of Obstetrics and Gynecology, Wright State University, ayton, Ohio 45409-2793, USA

## Abstract

**Objective:**

A long-term, follow-up study comparing mild and severe forms of endometriosis and their fecundability, on 28 women diagnosed with endometriosis in adolescence.

**Methodology:**

Twenty-eight patients were identified from a prospective cohort of 52 adolescents (ages 12 to 18 years) with operative diagnosis of endometriosis between July 1993 and December 1995. All patients presented with chronic pelvic pain unresponsive to conservative medical management. Diagnosis of pregnancy was made by sonographic identification of intrauterine pregnancy, positive serum human chorionic gonadotropin or pathological confirmation of products of conception. Patients were categorized as fertile or sub-fertile by having > 12 months of unprotected intercourse without conception. Follow-up was done for 8.6 years.

**Results:**

Staging of endometriosis was performed according to the American Society for Reproductive Medicine standards. Stage I = 14.3%; Stage II = 39.3%; Stage III = 42.8%; Stage IV = 3.6%. Fecundability rates in each stage were statistically significant: Stage I (75%), Stage II (55%), Stage III (25%), Stage IV (0%) (p < .05). Rates of spontaneous abortion were not statistically significant.

**Conclusion:**

In our cohort, even at the earliest point in the natural life cycle of endometriosis there is an inverse relationship between stage of disease at diagnosis and fecundability.

## Background

Recent studies [1] have given us more insight into the pathophysiology of endometriosis. Fragments of functional endometrium reflux through the fallopian tubes and reach the essentially hostile environment of the peritoneal cavity. Proteolytic activity, activated macrophages and natural killer cells all combine to degrade and digest the regurgitated tissue fragments. Occasionally whole fragments of endometrial tissue succeed in evading the peritoneal defense lines, perhaps by their sheer number, perhaps by an intrinsic defect in the defense system. Microtraumas to the peritoneum will expose the extracellular matrix; successful endometrial tissue implantation may occur, and the cyclic angiogenesis that occurs in the female reproductive tract, will allow survival and growth of the ectopic endometrial tissue. Then subsequent cyclic tissue remodeling and new tissue development will take place. The intricate interaction between the implant and the host tissue will eventually result in the condition called endometriosis. If the local immune system fails to repair its surface, then the development of the disease endometriosis characterized by pain, menstrual cycle disturbance and impaired fertility, will eventually prevail [2]. Endometriosis in early adolescence could be explained by the theory of metaplasia of embryonic mullerian remnants in an extrauterine location [3].

The role of adolescent endometriosis in the impairment of fertility in adult life has not been extensively evaluated.

The purpose of our study was to compare mild and severe forms of endometriosis and their fecundability in a cohort of 28 patients diagnosed with endometriosis in adolescence and followed for 8.6 years.

## Methods

The 28 patients of the present report were identified from a prospective cohort of 52 adolescents, assembled between July 1993 and December 1995. They were operatively diagnosed with endometriosis during their evaluation for chronic pelvic pain unresponsive to conservative medical management. The diagnosis of endometriosis was made through diagnostic laparoscopy and biopsies. No other interventions beside diagnosis were performed. The original staging of endometriosis was done at the time of the surgery, using the American Fertility Society revised standard classification system [4]. Confirmation of staging was done by review of the photographic material obtained during the diagnostic procedures by experienced gynecologic laparoscopists.

All patients received the same therapy consistent of continuous uninterrupted combined oral contraceptives for 6 months (Low Ovral^®^): 1 tablet orally per day, and Naproxyn Sodium 500 milligrams orally to be used occasionally for pain exacerbation.

In June 2002 after Institutional Review Board approval and informed consent was obtained, a telephone interview with each one of the patients was conducted to follow-up fertility assessment. All the patients were questioned regarding their general health status, their history of sexually transmitted diseases (STDs), use of contraception, other therapies utilized for the endometriosis, their desire for conception, fertility evaluation of their male partner, and their own fertility status.

Patients were considered to be fertile if they had received obstetric sonographic identification of an intrauterine pregnancy, had a positive serum beta human chorionic gonadotropin ≥ 25 International Units per milliliter or had pathologic confirmation of fetal or placental tissue after a spontaneous abortion or instrumental dilation and curettage. The patients were categorized as subfertile if they had more than twelve months of unprotected sexual intercourse during their fertile periods without conception.

Fecundability was defined as the probability of achieving a pregnancy with in one year period, with the presence of desire for conception.

Twenty-seven out of twenty-eight patients were successfully interviewed, and their clinical data was completely collected and processed. One patient transferred to Mexico and was not possible to be located. She was initially diagnosed with stage III disease.

The patient data was then tabulated and analyzed. Statistical analysis was performed using the Fisher exact chi square trend to compare fecundability and to evaluate differences between the groups. Statistical significance was taken as a P value of less than .05. Confidence intervals (95%) were calculated using the Modified Wald Method (The American Statistician) [5].

## Results

Their endometriosis was classified as: Stage I, four patients (14.3%); Stage II, eleven patients (39.2%), Stage III, twelve patients (42.8%) and Stage IV, one patient (3.6%).

Population characteristics regarding age at cohort entrance, age at follow-up, number of years trying to conceive, and age at diagnosis for endometriosis are displayed. (Table 1)

**Table 1:** Population Characteristics

Characteristics	Stage I	Stage II	Stage III	Stage IV	P value
Age at Cohort Entrance	14.3 ± 2.3	15.4 ± 2.9	15.2 ± 2.6	16	.06
Caucasian	3	9	10	1	.73
African American	1	2	2	0	.35
STD's	0	2	2	0	.50
Contraception Use	1	4	4	1	.08
Desire for Conception	3	9	10	-	.73
Male Partner's Evaluation	0	1	1	-	.12
Other Therapies Used	1	2	3	-	.25
Age at Stage of Endometriosis	15.2 ± 2.1	16.8 ± 2.7	16.7 ± 2.3	16.9	.535
Time Trying to Conceive (Month)	15.1 ± 4.5	12.8 ± 3.8	13.1 ± 4.2	-	.310
Age at Follow-up	22.3 ± 5.4	24.7 ± 4.6	25.3 ± 4.9	-	.125

Patient in stages I, II, III and IV were not statistically different regarding age, race, general health status, history of STDs, use of contraception, desire for conception, evaluation for infertility, male partner's fecundity evaluation, and therapies utilized for the treatment of their endometriosis. (Table 1)

**Figure 1:**
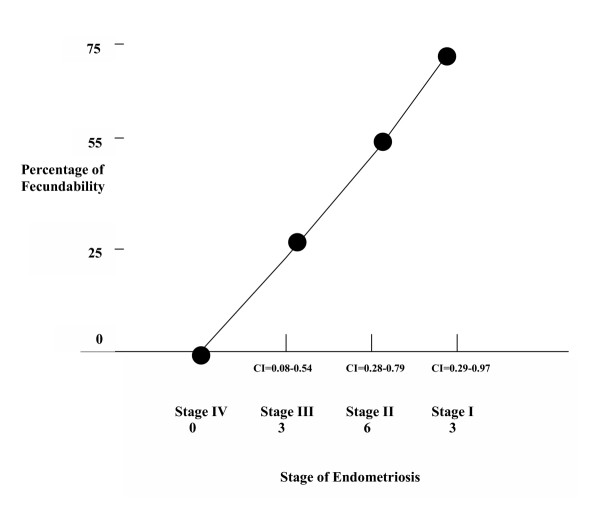
Relationship between stage of endometriosis and fecundability. (CI = Confidence Interval)

Fecundability rates in each stage were: Stage I = 3 patients (75%), Stage II = 6 patients (55%), Stage III = 3 patients (25%), Stage IV = 0 patients (0%) (P < .05) (Figure 1).

The rate of spontaneous abortions was not statistically significant among the stages:

Stage I = 1 patient (16.7%), Stage II = 1 patient (33.4%), Stage III = 1 patient (33.4%) (P < 0.05).

## Discussion

Endometriosis in adolescence is a cause of chronic pelvic pain and dysmenorrhea. The goal of therapy is to minimize pelvic pain and dysmenorrhea, primarily through long term medical therapy. Although endometriosis is associated with infertility, a clear causal relationship has yet to be established, except when adhesive disease is found [2].

The epidemiologic study of endometriosis presents researchers with unique Challenges. As a result, few well-designed studies have been published since the pathogenesis of endometriosis is still not yet fully understood [6].

Endometriosis is a progressive disease without a definitive cure. Therefore, adolescents with endometriosis require long-term medical management until they have completed their own fertility goal at childbearing age [6].

It is now well established that surgical management of endometriosis in the early stage of disease increases pregnancy rates (level I evidence based) [2]. Treatment of moderate to severe endometriosis also confers benefit although the evidence to support this treatment is using the United States Preventive Services Task Force classification of level II – III [2].

The above statements were not known nor applied to this cohort of adolescents since they were diagnosed with endometriosis between 1993 and 1995. The state of the art therapy for adolescence endometriosis during the referenced years was the use of oral contraceptive pills in a pseudo pregnancy fashion (continuous medication without withdrawal period) and the use of non-steroidal anti-inflammatory medications [7].

Our study, evaluating the fecundability of adolescents with endometriosis is unique because of the long follow-up 8.6 years. Also, the time elapsed between symptoms and diagnosis was relatively short. A recent study by Arruda et al. [8] showed a delay in diagnosis of endometriosis of 7.4 years for patients with pelvic pain.

Our findings, although the sample population is relatively small, confirm the well demonstrated fact observed in the adult population that there is an inverse relationship between endometriosis disease progression and fecundability [9]. Evidence supports surgical intervention, especially in stage 1 and 2 endometriosis to improve fecundity rates. In infertile women, laparoscopic resection or ablation of minimal and mild endometriosis practically doubles fecundity when compared to diagnostic laparoscopy alone [10]. Although a recent Italian study [11] was unable to show statistical improvement in birth rate after laparoscopic resection or ablation of endometriosis.

Our study suggests that the inverse relationship between stage of the endometriosis at diagnosis and fecundability seems to be present even at the earliest point in the natural life cycle of endometriosis.

## Conclusion

In our cohort study, even at the earliest point in the natural life cycle of endometriosis there is an inverse relationship between stage of disease at diagnosis and fecundability.
